# Supplementary material for: Identification of photoperiod-induced specific miRNAs in the adrenal glands of Sunite sheep (Ovis aries)
Source: Front Vet Sci. 2022 Jul 22;9:888207. doi: 10.3389/fvets.2022.888207 (PMC9354845; doi:10.3389/fvets.2022.888207)
Supplement: Supplementary file 1 [file Data_Sheet_1.ZIP › Supplementary file/miRDeep2 file.zip!/miRDeep2 file/Novel_Structure/LP42A2/Novel_514.pdf]

| | | |
|---|---|---|
| Provisional ID | : | NC_019483.2_36325 |
| Score total | : | 16.2 |
| Score for star read(s) | : | 3.9 |
| Score for read counts | : | 8.8 |
| Score for mfe | : | 1.9 |
| Score for randfold | : | 1.6 |
| Score for cons. seed | : | |
| Total read count | : | 29 |
| Mature read count | : | 27 |
| Loop read count | : | 0 |
| Star read count | : | 2 |

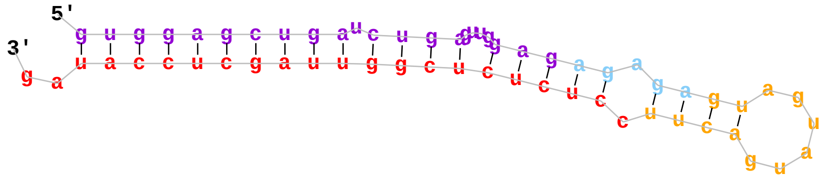


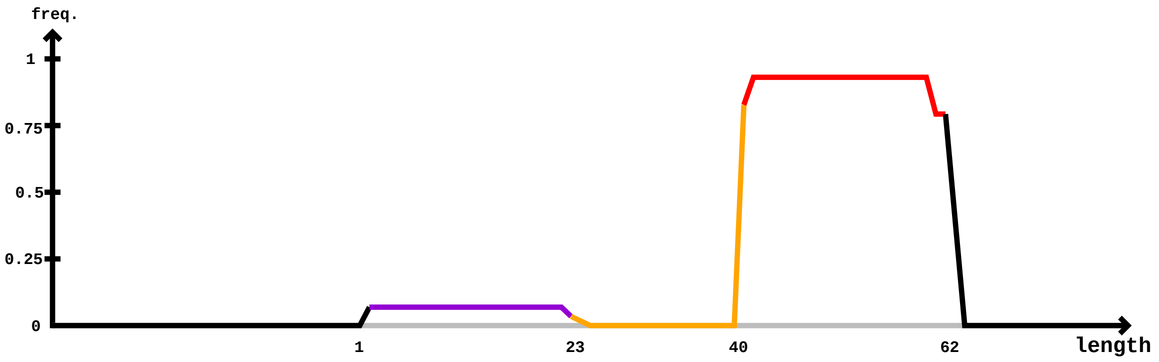


**Star** **Mature**

```
5'- uuuacucaguucacugaccgauugugccaguguggagcugaucugaguuggagagagaguaguaugacuuccucucucgguuagcuccauagauggcagaucagccuucu  -3'  obs
    uuuacucaguucacugaccgauugugccaguguggagcugaucugaguuggagagagaguaguaugacuuccucucucgguuagcuccauagauggcagaucagccuucu        exp
    .....((((....))))..((((.(((((.(((((((((((.((((....(((((.((((......)))).))))))))))))))))))))..)))))))))........   reads   mm   sample
    ...............................guggagcugaucugaguugga..........................................................   1       0    seq
    ...............................guggagcugaucugaguuggag.........................................................   1       0    seq
    ......................................................................ccucucucgguuagcuccau....................   4       0    seq
    ......................................................................ccucucucgguuagcuccauag..................   14      0    seq
    ......................................................................ccucucucgguuagcuccauaga.................   6       0    seq
    .......................................................................cucucucgguuagcuccauag..................   3       0    seq
```
